# Perceived Gaps in Oncologic Emergency Care for Patients with Cancer: A Qualitative Comparison of Emergency Medicine and Oncologist Physician Perspectives

**DOI:** 10.3390/cancers17050828

**Published:** 2025-02-27

**Authors:** Monica K. Wattana, Moira Davenport, Jason J. Bischof, Angela B. Lindsay, Nicholas R. Pettit, Jazmin R. Menendez, Kelsey Harper, Demis N. Lipe, Aiham Qdaisat

**Affiliations:** 1 Department of Emergency Medicine, The University of Texas MD Anderson Cancer Center, Houston, TX 77030, USAaqdaisat@mdanderson.org (A.Q.); 2 Department of Emergency Medicine, Allegheny General Hospital, Allegheny Health Network, Pittsburgh, PA 15212, USA; 3 Department of Emergency Medicine, The Ohio State University Wexner Medical Center, Columbus, OH 43210, USA; 4 Department of Emergency Medicine, Virginia Commonwealth University, Richmond, VA 23298, USA; angela.creditt@vcuhealth.org; 5 Department of Emergency Medicine, Indiana University, Indianapolis, IN 46202, USA; nrpettit@iu.edu; 6 Department of Emergency Medicine, Brown University, Providence, RI 02912, USA; 7 ProPharma Group, Raleigh, NC 27601, USA

**Keywords:** emergency, care, oncology, gaps, knowledge, system constraints, interdisciplinary care, cancer, oncologist, emergency medicine

## Abstract

**Objective:** Providing high-quality, safe, and consistent care for patients with cancer in the emergency department (ED) poses unique challenges. To better understand these challenges, we surveyed oncologists and emergency medicine (EM) physicians across five institutions to identify key areas for improvement in oncologic EM. **Methods:** In this multi-institutional, cross-sectional qualitative study, a semi-structured survey was administered to EM attending and resident physicians and medical and surgical oncologists across five institutions in 2023. We assessed the open-ended questionnaire responses using thematic analysis; codes were created and collated to generate initial themes. The themes were then reviewed according to specialty for coherence and non-repetition and finalized. **Results:** Of the 302 surveys accessed, 185 (61.3%) had complete responses. Three main domains of issues emerged: systems-based challenges, direct patient care-related issues, and knowledge gaps. The issues most frequently perceived by oncologist survey respondents were long delays in care (41%), variability in care (25%), and communication issues between the EM physician and oncologist (14%). The issues most frequently perceived by EM physician survey respondents were knowledge gaps in cancer therapeutics (40%) and in general oncologic emergencies (23%); physician comfort level (14%); the timing and/or location of initial discussions about goals of care (13%); and challenges with the follow-up process (12%). **Conclusions:** Incorporating an interdisciplinary approach to patient care in the ED, improved EM oncologic education, and the development of oncologic specialized EDs may enhance the quality, safety, and consistency of care for patients with cancer in the ED.

## Simple Summary

To better understand the challenges faced in treating cancer patients in emergency departments, a multi-institution survey of oncologists and emergency medicine physicians was carried out to identify the key challenges in treating cancer patients in emergency departments. Three core issue domains were recognized: system-based challenges, issues in patient care, and knowledge shortages. Adopting a collaborative approach to cancer patient care in the emergency department, enhancing emergency medicine education in oncology, and creating specialized oncology-focused EDs could improve the quality, safety, and consistency of care for cancer patients.

## 1. Introduction

Patients with cancer visit the ED approximately 3 million to 5 million times per year [1]. These patients’ visits comprise approximately 4% of all adult ED visits, a rate that is similar to those of patients with congestive heart failure (4%), cerebrovascular disease (3.7%), and chronic kidney disease (3.5%) [1,2]. Visits to the ED by patients with cancer are expected to increase as cancer therapeutics improve survivorship and early detection strategies allow for earlier diagnosis and treatment initiation [1,3].

Patients with cancer visit the emergency department (ED) more frequently than the general population and often present with chief complaints of higher acuity [4,5,6,7]. Although studies have shown that as many as half of these ED visits may be preventable [8], visits to the ED by patients with cancer result in higher admission rates, seven-day revisit rates, and resource utilization compared with visits by patients without cancer [1,5,9,10,11,12,13]. Providing high-quality, safe, and consistent care for patients with cancer in the ED poses unique challenges [10].

Currently, the quality of care that patients with cancer receive in the ED is not standardized and fluctuates widely among institutions. Factors such as extended wait and boarding times and the lack of established clinical pathways, validated decision tools, and physician familiarity with oncologic emergencies have been reported to contribute to the varying levels of the quality of care [12,13,14].

Our findings stem from an initial effort to help standardize oncologic emergency medicine education during residency training by performing an educational needs analysis by asking medical and surgical oncologists, emergency medicine (EM) attending, and resident physicians at multiple hospitals across the United States about knowledge gaps in oncologic EM education [15]. As participants identified more gaps beyond the focused scope of residency curriculum design, qualitative analysis was deemed necessary to uncover and better understand knowledge gaps in oncologic EM. We concentrate on the perspectives of emergency medicine physicians and oncologists, as these specialists are the primary stakeholders responsible for the care of patients in their respective practice environments. Previous studies have shown that communication gaps exist between outpatient and inpatient settings [16]. In at least one previous study, ED personnel were interviewed to identify ways to enhance the care of patients with cancer in the ED [17]; however, oncologists’ views on the ED management of their patients have yet to be documented.

Analyzing the similarities and differences in perspectives between these two key stakeholders enables a more thorough identification of crucial issues that, if addressed, can enhance cancer care in the ED. Since the ED often serves as the entry point for hospital admission, this will ultimately improve the continuity of oncologic care from the outpatient clinic to the ED and inpatient setting. The present study analyzed qualitative feedback obtained from a survey [15], asking medical and surgical oncologists and emergency medicine (EM) attending and resident physicians at multiple hospitals across the United States about knowledge gaps in oncologic EM.

## 2. Methods

### 2.1. Site Selection and Study Design

In this multi-institutional, cross-sectional, qualitative study, a survey was sent to EM attending and resident physicians and medical and surgical oncologists across five institutions: The University of Texas MD Anderson Cancer Center (Houston, Texas), Virginia Commonwealth University (Richmond, Virginia), Indiana University (Indianapolis, Indiana), Allegheny Health Network (Pittsburgh, Pennsylvania), and The Ohio State University Wexner Medical Center (Columbus, Ohio). The institutional review board of each institution approved the study. The authors are members of the Society of Academic Emergency Medicine Oncologic Emergencies Interest Group, and the survey sites correspond to the authors’ institutions. Based on previous similar studies [18,19,20], we estimated that 50 participants from each group (EM physicians and oncologists) would be needed to ensure sufficient data for thematic analysis and to reach code saturation.

### 2.2. Survey Design and Distribution

The Research Electronic Data Capture (REDCap) platform [21], which is hosted at each participating institution, was used to generate and distribute the survey using an invitation link sent to the participants’ email addresses to ensure consistency in data collection. The semi-structured survey included open-ended questions designed to enable respondents to freely express their concerns, feedback, and perceptions about gaps in the care of patients with cancer in the ED.

The questionnaire’s structure and development were described previously [15]. The survey questionnaires are listed in Table S1. Briefly, EM attending and resident physicians were asked, “What concerns do you have when caring for an oncology patient?” Medical and surgical oncologists were asked, “What concerns do you have when you send a patient to the ED?” Both EM physicians and oncologists were also given the opportunity to provide any additional feedback in a separate response related to the care of patients with cancer in the ED.

Convenience sampling was used to create a list of potential survey recipients that included EM attending and resident physicians and oncologists at each institution. By including five institutions across different states and surveying EM attending and resident physicians, as well as medical and surgical oncologists, we ensured the triangulation of the study. This approach allowed us to gather diverse perspectives and enhance the validity of our findings. A study member distributed the survey to these individuals via email invitation links at each site between October 2022 and September 2023. Two weeks after the survey was sent, each recipient who had not accessed the survey received an automated reminder about the survey through REDCap; if the survey remained inactive two weeks later, the recipient was sent a second, final reminder. The survey was deemed complete if respondents answered all the primary structured questions and at least one of the open-ended questions.

### 2.3. Data Analysis

Descriptive statistics (frequencies and percentages) were used to summarize the demographic characteristics of the survey respondents. Utilizing the content analysis approach, thematic analysis following the six-step process developed by Braun and Clarke was used to assess and investigate the open-ended questionnaire responses [22]. Inductive thematic analysis of all the responses was conducted with both semantic and latent approaches. To ensure inter-rater reliability, three investigators (MKW, MD, AQ) blindly created the codes; after which data codes were compared and finalized by consensus and collated to generate initial themes. The themes were then reviewed according to physician specialty for coherence and non-repetition and finalized in relation to all responses. Exploratory textual analysis with a word cloud was used to compare the frequencies of the identified codes between EM physicians and oncologists. A mind map was created to visualize the perceived gaps in care identified from the thematic analysis.

The descriptive statistics analyses were performed, and the word cloud was created using R software (version 4.2.3, The R Foundation, https://www.r-project.org, accessed on 2 January 2024).

## 3. Results

### 3.1. Respondent Characteristics

Of the 833 survey invitation links distributed across MD Anderson (*n* = 427, 51.3%), The Ohio State University (*n* = 176, 21.1%), Virginia Commonwealth University (*n* = 103, 12.4%), Allegheny Health Network (*n* = 96, 11.5%), and Indiana University (*n* = 31, 3.7%), 302 links (36.3%) were accessed. Of the 302 opened surveys, 185 (61.3%) were completed. Table 1 shows the characteristics of the survey respondents.

### 3.2. Survey Results Overview

**Figure 1:**
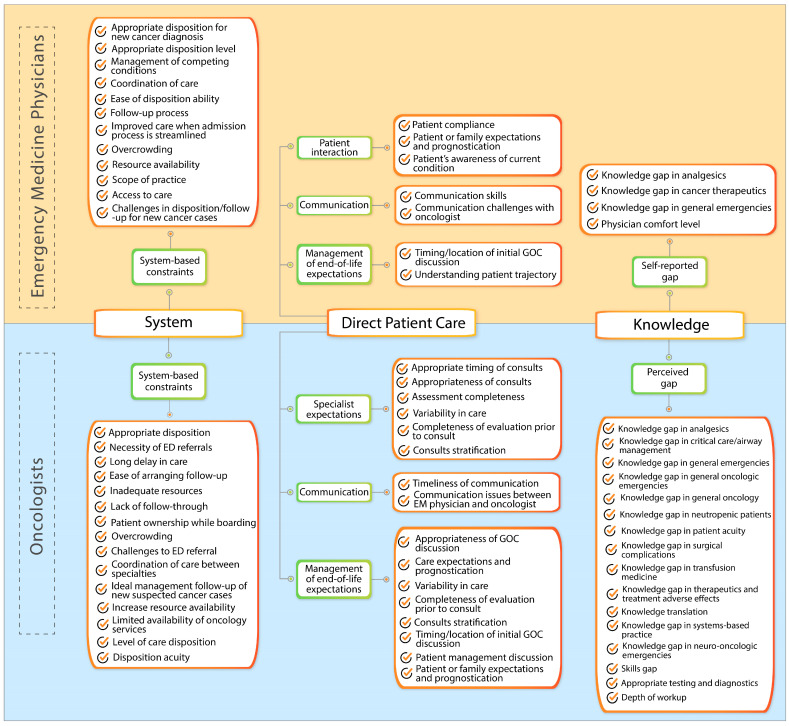
Mind map for the perceived gaps in care for patients with cancer in the emergency department (ED). GOC, goal of care.

EM physicians and oncologists expressed concerns about managing patients with cancer in the ED. An analysis of the themes and codes in both groups revealed three main domains: systems-based constraints, direct patient care-related issues, and knowledge gaps. Figure 1 lists the codes that fall under each domain for both the EM physician and oncologist groups.

Table 2 illustrates examples of quotations exemplifying each theme expressed by both respondent groups.

**Figure 2:**
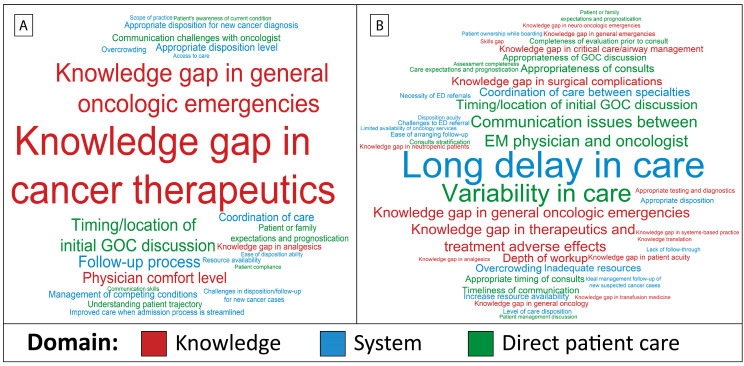
Word cloud analysis of the frequency of codes for the issues identified regarding the care of patients with cancer in the emergency department (ED). (**A**) Issues perceived by emergency medicine physicians. (**B**) Issues perceived by oncologists. GOC, goal of care. Code size represents the relative frequency of these terms based on the responses.

The relative frequency of issues expressed by oncologists and EM physicians is demonstrated in Figure 2. Figure 2A and Table S2 show the frequency of issues established by the feedback from EM physicians’ survey respondents. The two most frequently perceived issues were a knowledge gap in cancer therapeutics (*n* = 38, 40%) and a knowledge gap in oncologic emergencies (*n* = 22, 23%). The three next most common issues were physician comfort level (*n* = 13, 14%), the timing and/or location of an initial goal of care (GOC) discussion (*n* = 12, 13%), and challenges with the follow-up process (*n* = 11, 12%). Figure 2B and Table S3 show the frequency of issues established by oncologists’ survey respondents’ feedback. The three most frequently perceived issues were long delays in care (*n* = 37, 41%), variability in care (*n* = 23, 25%), and communication issues between EM physicians and oncologists (*n* = 13, 14%).

### 3.3. Systems-Based Constraints

#### 3.3.1. ED Referral Issues and Concerns During ED Workup

Patients with cancer occasionally present to the ED for further evaluation after a referral from their oncologist. Both EM physicians and oncologist respondents expressed concerns about the appropriateness of these referrals. EM physicians at multiple institutions questioned whether direct admission would better serve patient needs (6%) and alleviate ED overcrowding (3%). Additionally, some EM physicians questioned the necessity of extended ED-based evaluations (3%), saying that more advanced testing, such as magnetic resonance imaging or laboratory tests not routinely ordered in the ED, is required for the diagnosis or expedited management of certain oncologic emergencies and is outside the scope of EM practice; they noted that such testing also prolongs a patient’s stay in the ED. Lastly, EM physicians expressed concern that some EDs, particularly those in resource-limited areas (2%), may not be capable of such advanced testing.

Oncologists also expressed frustration that direct admission to the hospital was not always possible owing to issues such as overcrowding (8%) and inefficient admission processes (2%). The delay in the workup and treatment initiation due to prolonged waiting room times was also a major concern (41%).

#### 3.3.2. ED Boarding Concerns

Ownership of the care of patients with cancer boarding in the ED was a concern for both specialties; both EM physicians and oncologists said that once a patient is admitted, management decisions should be the purview of the admitting service. However, respondents also admitted that this viewpoint may not reflect reality, as EM physicians may continue to provide care while patients are waiting for a hospital bed or are waiting to be transferred to an outside facility to receive a higher level of care.

#### 3.3.3. ED Disposition Concerns

Both groups commented on the challenges associated with the disposition of patients with an existing cancer diagnosis and patients with a suspected new cancer diagnosis in the ED. For patients with an existing cancer diagnosis, both groups expressed concern about difficulties in arranging an expedited follow-up with oncology services. EM physicians were also concerned about ensuring that patients with a suspected new cancer diagnosis receive timely care (2%). Some EM physicians commented that outpatient management seemed less than ideal, given the perceived lack of access to timely outpatient oncology appointments (4%). Conversely, oncologists said that many patients with a suspected cancer diagnosis could be evaluated on an outpatient basis rather than during inpatient stays. Furthermore, oncologists expressed a desire for patients with cancer who are admitted to be placed in the appropriate service (e.g., bone marrow vs. solid tumor units).

### 3.4. Direct Patient Care-Related Issues

Each group identified three direct patient care-related issues. Two types of issues were the same for both groups: communication between EM physicians and oncologists and the management of end-of-life expectations. The third type of issue differed between the two groups: EM physicians had concerns about patient interactions, such as those regarding patient and/or family expectations about prognostication and awareness of the disease burden (4%), whereas oncologists had concerns about expectations for the level of care received by their patients in the ED, including concerns about the variability of care among EM physicians (25%) and concerns about the completeness of assessment before a consultation is requested (3%).

#### 3.4.1. Communication Concerns

EM physicians reported difficulties contacting their oncologist colleagues (5%), especially after hours, to notify them or to discuss patient management, especially if the oncologist was not in the hospital network. Oncologists commented on the extent of evaluation completed in the ED before they were contacted (9%). Most oncologists felt that consultations were requested prematurely and before a thorough workup was completed, limiting the effectiveness of the communication (3%). This concern was reported more frequently by surgical oncologists than by medical oncologists. Additionally, oncologists believed that EM physicians do not appropriately stratify the urgency of consultations and said that many of these evaluations could wait until the patient is admitted (8%). Oncologists also were concerned that when they attempted to communicate with EM physicians (5%), such as via phone call or notification in a patient’s chart, the communicated plans were not received by the practitioner or carried out. Oncologists’ responses also indicated that treatment plans for patients with cancer may require the coordination of multiple specialties (9%), but that communication among these specialties can be difficult when such patients are in the ED (14%).

#### 3.4.2. End-of-Life Expectation Concerns

Both EM physicians and oncologists mentioned difficulties related to end-of-life discussions. EM physicians said that many oncologists delay this topic during outpatient appointments, leaving the burden of the discussion to EM providers (14%). Interestingly, oncologists expressed a desire to have more end-of-life discussions in the outpatient setting (11%).

#### 3.4.3. Disease Burden Discussion Concerns

Many EM physicians expressed concerns about managing the patient’s and/or their family’s expectations, particularly those regarding disease progression and prognostication (4%), as EM physicians felt that most of these discussions were not within the scope of EM practice.

#### 3.4.4. Concerns About the Variability of Care Between EM Physicians

Oncologists expressed concerns about the variability in the treatment of oncologic emergencies in the ED (25%), indicating that, besides the initiation of communication, the depth of workup and management may differ among physicians within the same institution. Oncologists also cited concerns about “survivorship bias”, noting that some patients, simply because they have had cancer, receive in-depth, but potentially unnecessary, imaging and extensive laboratory workup. Oncologists also expressed frustration about the minimal workup performed before specialist services or admissions are contacted, saying that they often prefer to have complete laboratory and imaging results before being consulted (3%).

### 3.5. Knowledge Gaps

Perceived gaps in EM physicians’ medical knowledge were concerns of both specialties. EM physicians repeatedly mentioned a need for additional training on cancer therapeutics (40%), particularly newer immunologic agents and their adverse effects. Oncologists were concerned about EM physicians’ knowledge of general oncologic care (3%), oncologic emergencies (12%), oncologic surgery complications (9%), transfusion medicine (1%), and the acuity of cancer-related ED presentations (3%). Both groups reported the need for increased EM physicians’ education about the role of analgesics in oncology, particularly because these agents may interact with cancer therapies.

## 4. Discussion

The results of our study emphasize that a one-size-fits-all approach is not effective for patients with cancer receiving acute care in the ED; strategies, processes, and algorithms used for the general population in the ED are likely not ideal for patients with cancer. Unlike for patients with other disease processes, the development of universal guidelines and standardization of diagnosis and management for patients with cancer in the ED has yet to occur. Additionally, cancer–hospital networks and dedicated oncologic EM curriculums have not been widely adopted. We suggest the following three interventions to help address concerns identified in our survey and improve the quality of care that patients with cancer receive in the ED: using an interdisciplinary approach to patient care, increasing the oncologic EM knowledge of EM physicians, and creating specialized EDs for cancer care.

### 4.1. Interdisciplinary Approach to Patient Care

EM physicians play a critical role in cancer care [23,24]. However, the care provided in the ED is not provided in isolation but in collaboration with hematologists, oncologists, radiation oncologists, palliative care providers, social workers, and other medical care teams [25,26]. Coordinating this care with the EM physician serving as the central facilitator can help address multiple issues identified in our study.

Patients with cancer often visit the ED at the request of their oncologist or treatment team [13,27]. Timely and clear communication between the patient’s oncologists or other specialists with the ED before initiating testing and imaging for patient evaluation may offer key information, such as potential adverse effects, and enhance medical management, thereby improving patient outcomes. Similarly, communication between the oncologist and the patient or their family may help manage their expectations for receiving care in the ED. Managing patient expectations is particularly important given the current state of inpatient boarding and long wait times in EDs across the country [28,29]. Offering reassurance that a diagnostic strategy and treatment plan have already been formulated may ease the anxiety of the patient and improve their satisfaction with the care they receive.

EM physicians routinely have GOC discussions with patients. Successful GOC discussions allow for the appropriate triage of hospital resources and avoid decisions that ultimately may not be congruent with a patient’s quality-of-life goals [30,31]. If a patient’s condition is rapidly declining and there are concerns for impending mortality, the onus to facilitate end-of-life discussions often falls on the EM physician. Often, this situation is not ideal. The patient and EM physician do not have a preexisting relationship, making these conversations even more difficult. If time and the patient’s condition permit, including the patient’s oncologist or palliative care services can help to facilitate GOC discussions while the patient is in the ED. Such discussions could occur in person if the clinician is onsite or, although less ideal, over the phone. Including the patient’s oncologist or palliative services in GOC discussions may not be feasible in many EDs. This is especially true if the oncologist does not have privileges in the institution affiliated with the ED, if the conversation must occur after clinic hours, or if supportive and palliative services are not available in the ED. Ultimately, we urge our oncologist colleagues to have GOC discussions with patients with cancer on a more routine basis during clinic visits. Prior discussions about these topics help normalize GOC conversations during ED visits.

### 4.2. Increasing the Oncologic EM Knowledge of EM Physicians

A standardized oncologic EM curriculum for EM physicians does not exist; there is no gold-standard didactic reference for graduate medical education programs. The quantitative results of the survey used in the present study, which were reported previously, identified key topics that can be used to develop an oncologic EM curriculum that will enhance the foundational knowledge of EM resident physicians [15]. Additionally, the need for oncologic EM learning continues after residency, as practicing EM attending physicians responding to our survey also requested increased education and more methods of oncologic EM knowledge acquisition.

As new cancer therapeutics are adopted and survivorship increases, the scope of oncologic EM knowledge will continue to expand. Fellowships, niche positions, and focused training opportunities in EM have been created to help promote awareness and improve specialized education [32]. Increasing expertise through a fellowship in oncologic EM may be helpful. Currently, only two US institutions, MD Anderson and The Ohio State University, offer an oncologic EM fellowship. We also support the formation of oncologic EM interest groups within EM societies and cross-institutional educational and research collaborations to promote increased awareness of oncologic emergencies. Currently, there is no gold-standard oncologic EM curriculum that accurately reflects the most common and severe ED presentations and up-to-date adverse effects from treatment therapeutics, and the development and dissemination of such a curriculum will also help lessen current knowledge gaps [33].

### 4.3. Specialized EDs for Cancer Care

Given the increasing number of patients with cancer who require complex care, there is a need to increase the number of EDs associated with cancer treatment centers [34]. EDs can obtain certifications for conditions such as stroke and acute coronary syndrome, and evidence has shown that timely transfers to these specialized centers enhance patient outcomes [35,36,37]. Theoretically, oncology-specialized EDs would be better able to handle common oncologic emergencies through an increased availability of specialized oncology services, such as interventional radiology, surgical oncology, and radiation oncology; routinely stocked medications for common adverse effects associated with cancer treatment; and an increased availability of pain management specialists and palliative care services to better care for pain-related concerns.

While ideal, immediately allocating resources and finances to create more ED-specific cancer centers may not be practical. We also support establishing guidelines and criteria for ED certifications in cancer care as a more achievable option. ED certifications already exist, such as for the geriatric population, and have a concordant department accreditation developed by the American College of Emergency Physicians [38,39,40]. Geriatric ED specialization focuses on recognizing how the geriatric ED population is distinct from the general population and creating measurable criteria organized into accreditation tiers that incorporate resources that often already exist and can be easily integrated into current operations for all types of ED practice environments within an academic, community, and rural setting. Examples of initiatives that meet geriatric ED accreditation include taking measures to reduce fall risk within the ED, providing methods such as whiteboards and headphones to improve communication for hard-of-hearing patients, and enhancing disposition methods tailored to geriatric patients so that they understand how and when to take new medications [41,42,43,44].

Likewise, developing a certification for specialized cancer care is an ideal endeavor for improving the quality of care for the cancer patient population. Certification can be tiered, similar to geriatric accreditation, and can include essential gaps identified in our study regarding education, system-based practice, and communication. Initiatives that do not require additional staffing or excessive financial constraints and are adaptable to a diverse ED practice environment can be considered such as a means of alerting ED physicians within the electronic medical record of when a patient is actively on immune checkpoint inhibitor therapy so that side effects may be considered, improving coordination of hospice initiation through the ED, and easing the process of communication between oncologists and ED physicians [45]. However, in the meantime, identifying in-network cancer hospitals and improving transfer processes may also help address the concerns identified in the present study.

### 4.4. Geographical and Practice Setting Variability: Implications for Generalizability and Future Research

While this study was conducted at hospitals across various geographic locations, allowing for consideration of differences in geographical practice parameters, the survey was mainly completed by academic physicians. Community-based physicians might have different experiences and concerns, which could be even more pronounced and challenging in settings with fewer resources [12,46,47]. Follow-up evaluations that examine distinct factors affecting patients with cancer within various medical systems and patient populations in regional, community, and smaller non-academic centers compared to academic institutions must be analyzed in greater depth to assess the generalizability of our results.

Although EM physicians in various settings are likely to value education on cancer therapeutics, further research is needed to design educational initiatives that address the distinct needs of each practice environment. Our analysis identified gaps across multiple domains grouped into systems-based and patient care-related concerns. Addressing further research directions based on our identified gaps will have wide-reaching benefits that will improve the care of patients with cancer in the ED. These projects can focus on impacting change within a single hospital or hospital system through targeted quality improvement projects, such as those to help facilitate care and knowledge transfer between patients, consultants, and ED providers, to public health initiatives that promote the improvement of cancer care in the ED, addressing educational barriers to ED care by patients with cancer, which impact broader national and societal levels.

### 4.5. Limitations

The study had some limitations that should be considered. One limitation is the overrepresentation of academic physicians in the survey responses, which may not reflect the experiences of community-based physicians. Future research should consider including a more diverse sample of practice settings to offer a fuller picture of the challenges faced by EM physicians in different settings. Another significant limitation of the study is the relatively low access and response rates. Given the time-pressured schedules of ED physicians and oncologists, this may have contributed to the low response rate. Despite verifying that email addresses were correct and adjusting the date and time for reminders, the demanding nature of their work likely impacted their ability to participate in the survey. Finally, this study’s findings are based on responses from 185 participants, accounting for only 22% of the survey invitations distributed. Consequently, the data may not fully represent the perspectives of the entire workforce of ED physicians and oncologists. Additionally, the views expressed in the survey may not reflect the majority opinion, as only a small proportion of respondents highlighted these issues.

## 5. Conclusions

From their initial cancer diagnosis, patients with cancer present to the ED for cancer-related complications, adverse effect management, and conditions unrelated to cancer. As for all patients presenting to the ED for acute care, EM physicians are expected to provide high-quality and consistent care for patients with cancer, and doing so for this patient population is challenging. Our study identified three main domains for improving the care of patients with cancer in the ED: systems-based issues, patient care-related interactions, and knowledge gaps. Efforts focusing on addressing concerns within these domains may help to standardize and improve the care of patients with cancer presenting to the ED for acute care.

## Figures and Tables

**Table 1:** Participant characteristics.

Characteristics	*n* (%)
Total	185
Specialist	
Oncologist	91 (49.2)
Emergency medicine physician	
Attending	71 (38.4)
Resident	23 (12.4)
Practice setting	
Academic	148 (80.0)
Cancer hospital	21 (11.4)
Academic/community	13 (7.0)
Community	3 (1.6)
Years in specialty	
≤10	89 (48.1)
11–20	47 (25.4)
21–30	31 (16.8)
>30	18 (9.7)
Oncologic specialty *	
Solid tumor oncology	21 (23)
Hematologic oncology	41 (45)
Surgical oncology	14 (15)
Other or unspecified	15 (16)
Percentage of patients with cancer who require ED services *†	
1–10%	55 (62)
11–30%	25 (28)
>30%	9 (10)
Percentage of patients with cancer ‡	
1–10%	39 (41)
11–30%	40 (43)
>30%	15 (16)
Completed an oncologic emergency medicine rotation §	
No	20 (87)
Yes	3 (13)

Abbreviation: ED, emergency department. * Answered by only oncologists (*n* = 91). † Missing data for 2 respondents. ‡ Answered by only emergency medicine attending and resident physicians (*n* = 94). § Answered by only emergency medicine resident physicians (*n* = 23).

**Table 2:** Selected quotations exemplifying the main issues.

Issue	Selected Quotation		
**Emergency Medicine Physicians**			
Knowledge gap in cancer therapeutics	“Keeping up with all the advances—many of the treatments didn’t exist when I trained (even though it was not that long ago).”	“Not knowing all the drugs, the side effects, concern about starting a treatment that will [affect] the inpatient [team’s] ability to do diagnostics.”	“So many new treatments - hard to keep updated on possible complications.”
Knowledge gap in general oncologic emergencies	“Differentiating cancer-related etiology vs. something else emergent I should be worried about (“e.g.,” is your dyspnea from your primary cancer vs. chemo side effect vs. immunocompromised [pneumonia] vs. [pulmonary embolism] vs. atypical [acute coronary syndrome]).”	“Little education on managing cancer specific patient concerns/presentations.”	“Multiple simultaneous emergencies at once. Mimics of other illness. Contraindications to some standard treatment such as anticoagulation for pulmonary embolism or [deep vein thrombosis] because of thrombocytopenia or brain metastases. Refractory to symptom management.”
Physician comfort level	“Given their rarity and that our center is not the main center for [oncologic] emergencies I am less comfortable than many other emergencies.”		
Timing and/or location of initial goals of care discussion	“I am not impressed with most of these oncologists’ communication skills. Patients come into the [emergency department] with unrealistic expectations of survival and cure. The oncologist has not communicated to the patient and family the true inevitable outcome of their cancer. Also, patients arrive in the [emergency department] with end stage cancer and the oncologist has not discussed end of life care or hospice with the patient! Why does [this] fall upon the emergency department staff to discuss the sensitive, critical, and time-intensive issues with a patient we just met? The oncology team should have addressed end of life care with the patient and family in the clinic.”	“I don’t ‘routinely’ have end-of-life discussions with cancer patients, but I often do. However, when I do I am consistently surprised that these conversations have not been initiated by the patient’s oncology providers.”	
Issues with follow-up process	“Much emergency care is due to lack of timely care for many newly diagnosed patients with cancer, especially those with lack of financial resources.”		
**Oncologists**			
Long delay in care	“Long waiting time, not being called in a timely manner.”	“Prolonged time to first MD [physician] visit, long stay in the [emergency department], delayed time to [intravenous] antibiotics.”	“Standard delays and frustrations that are common to all [emergency departments]. In our neutropenic [patients], also want to minimize exposure to other patients with potential infections.”
Variability in care	“Inconsistent evaluation algorithm. Some have extensive evaluation prior to calling primary service and some have almost no assessment prior to calling primary service.”	“Different level of comfort of [emergency department] physicians when diagnosing and managing [immune-related adverse events].”	“I am often consulted by the emergency medicine group and there’s significant variability in the care provided. Certain providers are prone to consult services without seeing patients, prior to any work up and prior to the patients being roomed which often leads to the consulting services working up the patients and issues even if unrelated to their specialty.”
Communication challenges between the emergency medicine physician and the oncologist	“It does not seem like the [emergency department] staff look at our notes. I usually put a note in or detail what is going on/concerns in my last progress note, but it seems those are ignored.”	“I would find it helpful if the [emergency department] attending called me (at least during daytime) when the patient arrives so I can help guide them about work up, etc. They usually notify us of the admission after the patient has already arrived.”	“With explosion of new therapies with different sometime unpredictable side effect profiles, communication will expedite needed work-up.”

## Data Availability

The original contributions presented in this study are included in the article/Supplementary Material. Further inquiries can be directed to the corresponding author.

## Supplementary Materials

The following supporting information can be downloaded at: https://www.mdpi.com/article/10.3390/cancers17050828/s1, Table S1: General and specialty-specific survey questionnaire. Table S2. List and frequency of issues expressed by EM physicians (*n* = 94). Table S3. List and frequency of issues expressed by oncologists (*n* = 91).
