# Supplementary material for: Perceived Gaps in Oncologic Emergency Care for Patients with Cancer: A Qualitative Comparison of Emergency Medicine and Oncologist Physician Perspectives
Source: Cancers (Basel). 2025 Feb 27;17(5):828. doi: 10.3390/cancers17050828 (PMC11899144; doi:10.3390/cancers17050828)
Supplement: Supplementary file 1 [file cancers-17-00828-s001.zip › cancers-3440178-supplementary.pdf]

cancers

MDPI

Perceived Gaps in Oncologic Emergency Care for Patients with Cancer: A Qualitative Comparison of Emergency Medicine and Oncologist Physician Perspectives

Supplemental data

Table S1. General and specialty-specific survey questionnaire.

Question	Field/options
General	
What is your specialty?	Oncology<br>Emergency medicine attending physician<br>Emergency medicine resident
What is your practice setting?	Academic or training program<br>Community<br>Academic and Community<br>Cancer-Specific Hospital
Years in current specialty	0-4<br>5-10<br>11-20<br>21-3-<br>31-40<br>41+
Questions if answered "Oncology" for the specialty question	
What is your oncologic specialty?	Free text
Do your patients ever require care in an emergency department (ED)?	No<br>Yes
If yes for the above question, what percentage of your patients require ED services per month?	1-10%<br>11-30%<br>31-50%<br>51-75%<br>76-100%
What concerns do you have when you send a patient to the ED?	Free text
Please provide any additional feedback related to the care of oncology patients in the ED.	Free text
Questions if answered "Emergency medicine resident" for the specialty question	
What is your postgraduate year?	Free text
Have you completed an oncologic emergency medicine rotation?	No<br>Yes

Questions if answered either "Emergency medicine attending physician" or "Emergency medicine resident" for the specialty question	
Do you see cancer patients in your emergency department (ED)?	No<br>Yes
What percentage of your patients are cancer patients in a given month?	1-10%<br>11-30%<br>31-50%<br>51-75%<br>76-100%
What concerns do you have when caring for an oncology patient?	Free text
Please provide any additional feedback related to the care of oncology patients in the ED.	Free text

Table S2. List and frequency of issues expressed by EM physicians (*n* = 94).

Code	Frequency	Percentage
Knowledge gap in cancer therapeutics	38	40
Knowledge gap in general oncologic emergencies	22	23
Timing/location of initial GOC discussion	13	14
Follow-up process	12	13
Physician comfort level	11	12
Coordination of care	7	7
Appropriate disposition level	6	6
Communication challenges with oncologist	5	5
Management of competing conditions	5	5
Appropriate disposition for new cancer diagnosis	4	4
Knowledge gap in analgesics	4	4
Patient or family expectations and prognostication	4	4
Understanding patient trajectory	4	4
Improved care when admission process is streamlined	3	3
Overcrowding	3	3
Challenges in disposition/follow-up for new cancer cases	2	2
Resource availability	2	2
Access to care	1	1
Communication skills	1	1
Ease of disposition ability	1	1
Patient compliance	1	1
Patient's awareness of current condition	1	1
Scope of practice	1	1

Table S3. List and frequency of issues expressed by oncologists (*n* = 91).

Code	Frequency	Percentage
Long delay in care	37	41
Variability in care	23	25
Communication issues between EM physician and oncologist	13	14
Knowledge gap in general oncologic emergencies	11	12
Knowledge gap in therapeutics and treatment adverse effects	11	12
Timing/location of initial GOC discussion	10	11
Coordination of care between specialties	8	9
Depth of workup	8	9
Knowledge gap in surgical complications	8	9
Appropriateness of consults	7	8
Overcrowding	7	8
Inadequate resources	6	7
Appropriate timing of consults	5	5
Appropriateness of GOC discussion	5	5
Knowledge gap in critical care/airway management	5	5
Timeliness of communication	5	5
Increase resource availability	4	4
Appropriate disposition	3	3
Completeness of evaluation prior to consult	3	3
Knowledge gap in general oncology	3	3
Knowledge gap in patient acuity	3	3
Appropriate testing and diagnostics	2	2
Care expectations and prognostication	2	2
Challenges to ED referral	2	2
Consults stratification	2	2
Ease of arranging follow-up	2	2
Knowledge gap in general emergencies	2	2
Knowledge gap in neutropenic patients	2	2
Level of care disposition	2	2
Necessity of ED referrals	2	2
Assessment completeness	1	1
Disposition acuity	1	1
Ideal management follow-up of new suspected cancer cases	1	1
Knowledge gap in analgesics	1	1
Knowledge gap in neuro-oncologic emergencies	1	1
Knowledge gap in systems-based practice	1	1
Knowledge gap in transfusion medicine	1	1
Knowledge translation	1	1
Lack of follow-through	1	1
Limited availability of oncology services	1	1
Patient management discussion	1	1
Patient or family expectations and prognostication	1	1
Patient ownership while boarding	1	1
Skills gap	1	1
